# Nucleotide Sequence-Homology-Independent Breakdown of Transgenic Resistance by More Virulent Virus Strains and a Potential Solution

**DOI:** 10.1038/srep09804

**Published:** 2015-04-27

**Authors:** Yi-Jung Kung, Bang-Jau You, Joseph A. J. Raja, Kuan-Chun Chen, Chiung-Huei Huang, Huey-Jiunn Bau, Ching-Fu Yang, Chung-Hao Huang, Chung-Ping Chang, Shyi-Dong Yeh

**Affiliations:** 1 Department of Plant Pathology, National Chung Hsing University, Taichung, Taiwan, R.O.C.; 2 NCHU-UCD Plant and Food Biotechnology Center, National Chung Hsing University, Taiwan, R.O.C.; 3 Department of Chinese Pharmaceutical Sciences and Chinese Medicine Resources, China Medical University, Taichung, Taiwan, R.O.C.; 4 Agricultural Biotechnology Center, National Chung Hsing University, Taichung, Taiwan, R.O.C

## Abstract

Controlling plant viruses by genetic engineering, including the globally important *Papaya ringspot virus* (PRSV), mainly involves coat protein (CP) gene mediated resistance via post-transcriptional gene silencing (PTGS). However, the breakdown of single- or double-virus resistance in CP-gene-transgenic papaya by more virulent PRSV strains has been noted in repeated field trials. Recombination analysis revealed that the gene silencing suppressor HC-Pro or CP of the virulent PRSV strain 5-19 is responsible for overcoming CP-transgenic resistance in a sequence-homology-independent manner. Transient expression assays using agro-infiltration in *Nicotiana benthamiana* plants indicated that 5-19 HC-Pro exhibits stronger PTGS suppression than the transgene donor strain. To disarm the suppressor from the virulent strain, transgenic papaya lines were generated carrying untranslatable 5-19 HC-Pro, which conferred complete resistance to 5-19 and other geographic PRSV strains. Our study suggested the potential risk of the emergence of more virulent virus strains, spurred by the deployment of CP-gene-transgenic crops, and provides a strategy to combat such strains.

The coat protein (CP)-mediated resistance strategy pioneered by Beachy and coworkers^1^,^2^ to control plant viruses through genetic engineering has been widely used to develop commercialised crops, including CP-transgenic squash and papaya^3^. Originally, CP-transgenic resistance was proposed to occur via protein-mediated interference with uncoating or by trapping the viral RNA, thereby inhibiting the replication of related invading viruses^4^. However, it was later found out that transgenic lines that do not accumulate transcript and protein from the transgene display even higher degrees of resistance, indicating that resistance occurs at the RNA level^5^. This RNA-mediated resistance is based on post-transcriptional gene silencing (PTGS), a host defence response to RNA^6^,^7^,^8^,^9^. Host RNA polymerase can target foreign RNA transcribed from a transgene and synthesise dsRNA that is recognised and cleaved by Dicer-like ribonucleases into 21-24 nt transgene-derived short-interfering RNAs (siRNAs)^10^,^11^. These siRNAs are loaded into ARGONAUTE1 (AGO1), a core protein of the RNA-induced silencing complex (RISC), which unwinds and retains the complementary siRNAs to guide the AGO endonuclease to target and cleave related RNAs in a sequence-homology-dependent manner^12^. Thus, PTGS-mediated transgenic resistance depends on the homology between a transgene and the corresponding viral genome^13^.

Because dsRNA intermediates are formed during the replication of plant RNA viruses, PTGS represent a major host response against invasion by RNA viruses. To counteract these host defences, plant viruses have evolved specialised proteins, such as the helper component-proteinase (HC-Pro) of potyvirus^14^ and the 2b protein of cucumovirus^15^, that suppress PTGS by compromising the components of the host gene-silencing pathway^16^.

Papaya (*Carica papaya* L.) is an important fruit crop in tropical and subtropical areas. The aphid-borne ss(+)RNA potyvirus *Papaya ringspot virus* (PRSV) is the main obstacle to large-scale commercial production of this fruit^17^. The lack of naturally resistant sources for use in conventional breeding, as well as the ineffectiveness of control measures such as agricultural practices for repelling aphid vectors and cross-protection from mild PRSV strains, necessitate the generation of transgenic resistance^18^. In Hawaii, Gonsalves and co-workers initiated the development of PRSV CP-transgenic papaya in the late 1980s. The CP-transgenic resistance of the resulting SunUp cultivar^17^ is triggered by PTGS and confers a high degree of resistance to PRSV Hawaii strain HA^19^. In 1998, the SunUp and Rainbow papaya cultivars were deregulated and approved for commercial application in the United States^17^, representing the first successful case of the cultivation of a transgenic fruit tree. However, the high genetic divergence of CP genes from other geographic PRSV strains^17^,^18^ limits the application of these two CP-transgenic cultivars in regions outside of Hawaii.

We previously used the CP coding sequence and contiguous 3'-untranslated region (CP-3'UTR) of a native PRSV strain, YK, to generate PTGS-based transgenic resistance using the papaya cultivar Tainung No. 2 in Taiwan^20^. The resultant YK CP-3'UTR transgenic lines confer a high degree of broad-spectrum resistance to different geographic strains from Taiwan (YK), Hawaii (HA), Thailand (TH) and Mexico (MX)^21^. However, the infection of these YK CP-3'UTR transgenic papaya lines by a different potyvirus *Papaya leaf-distortion mosaic virus* (PLDMV) during field trials^22^ necessitated the development of double-virus-resistant transgenic papaya lines that carry a chimeric untranslatable transgene comprising parts of the PLDMV CP and PRSV YK CP-3'UTR sequences^23^. Nevertheless, during repeated field trials over 4 years, both PRSV single-virus-24 and PRSV+PLDMV double-virus-resistant lines were found to be susceptible to a more virulent strain, PRSV 5-19^23^. The emergence of more virulent virus strains represents a serious threat to the application of CP-transgenic papaya cultivars resistant to either PRSV or PRSV+PLDMV.

The YK CP-3'UTR transgenic papaya lines exhibit broad-spectrum resistance to PRSV strains whose CPs share >89% nucleotide (nt) sequence identity^21^,^24^ with the transgene, including the different geographic strains TH (Thailand), HA (Hawaii) and MX (Mexico)^21^,^24^. However, PRSV 5-19 is able to overcome the single- or double-virus transgenic resistance despite the significantly higher nucleotide identity of its CP/3'UTR compared with YK (96/98%)^24^, implying that the resistance-breaking ability of PRSV 5-19 is not correlated with the level of sequence divergence between its CP-3'UTR and that of the transgene. Previously, Mäki-Valkama^25^ reported that although potato lines containing the P1 gene of PVY^O ^(a common strain) are resistant to PVY^O^, they are not resistant to PVY^N ^(a necrotic strain), despite complete sequence identity between the P1 genes of PVY^O^ and PVY^N^. Such anomalous, homology-independent breakdown of PTGS-mediated transgenic resistance by a more virulent strain suggests the involvement of viral elements that suppress PTGS-mediated resistance and may shut off the RNA silencing mechanism.

In this study, the mechanism of the homology-independent breakdown of CP-transgenic resistance by virulent strain 5-19 was investigated by analysing the reactions of the PRSV single-virus- and PRSV+PLDMV double-virus-resistant lines to the transgene donor strain YK, the resistance-breaking strain 5-19, and their recombinants. The results of the recombinant analysis indicated that 5-19 HC-Pro, a PTGS suppressor^15^,^26^, is responsible for the resistance-breaking ability of 5-19. Gene silencing suppression assays performed using transient expression in *Nicotiana benthamiana* verified that 5-19 HC-Pro exerts strong gene-silencing suppression than YK HC-Pro. Thus, the virulent strain 5-19 may have emerged in conventional plants and been inoculated into transgenic trees via aphid-mediated transmission. The stronger ability of this strain to suppress PTGS allows it to overcome CP-transgenic resistance in a sequence-homology-independent manner. To counter the threat of the 5-19 strain, transgenic papaya lines carrying an untranslatable 5-19 HC-Pro construct were developed. The resulting HC-Pro-transgenic papaya lines are resistant to the virulent strain 5-19, as well as to other geographic PRSV strains, suggesting that this approach may solve the problem of the emergence of more virulent strains from CP-transgenic lines.

## Results

### CP-transgenic resistance can be overcome in a nucleotide sequence-homology-independent manner

**Figure 1:**
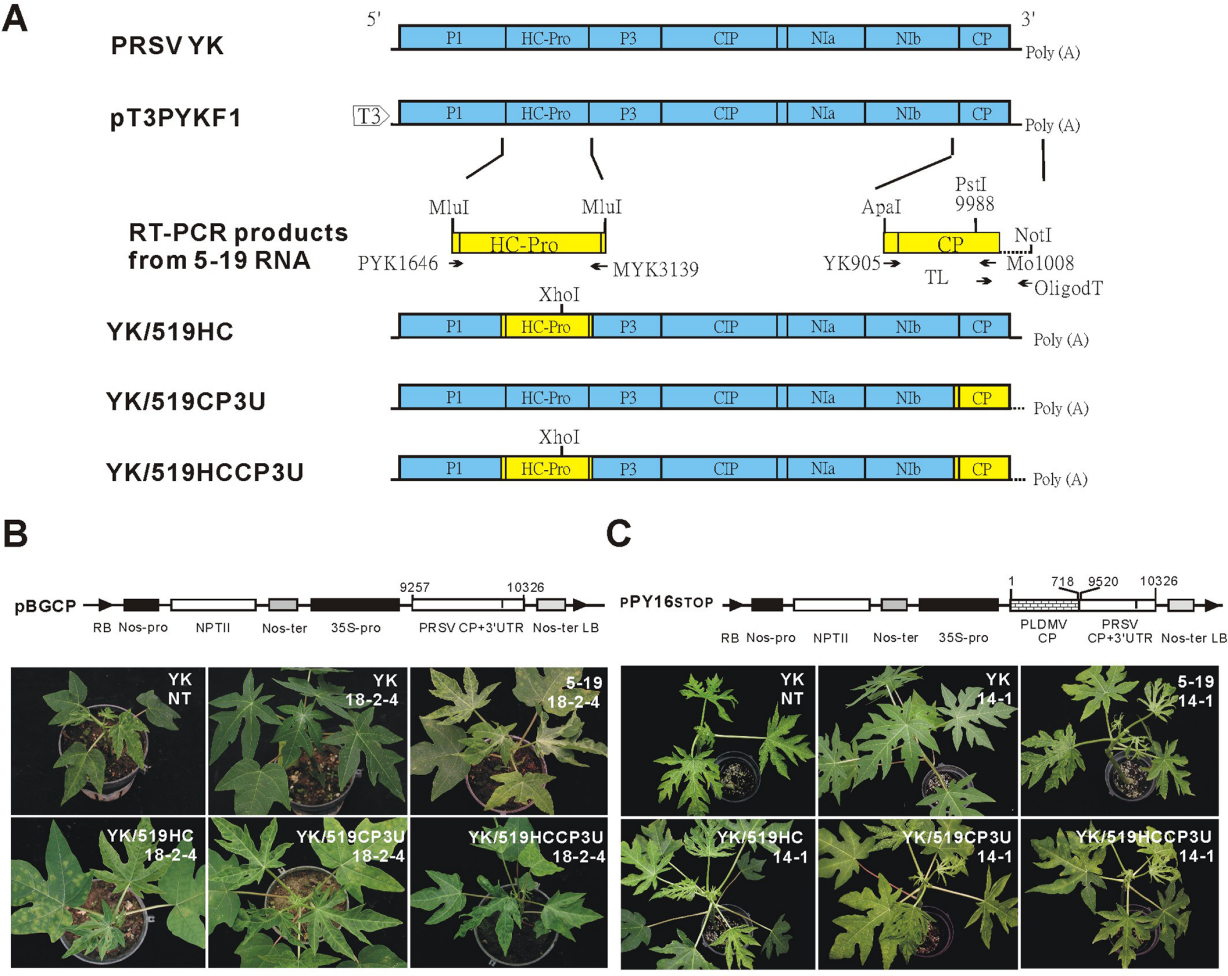
Recombination analysis to study the ability of helper component protease (HC-Pro) and coat protein (CP) from the *Papaya ringspot virus* (PRSV) resistance-breaking strain 5-19 to overcome single-virus or double-virus resistance in CP-transgenic papaya lines. **(A)** Construction of recombinants carrying heterologous HC-Pro or/and CP from the resistance-breaking strain 5-19 using the *in vitro* infectious cDNA from the non-breaking strain YK as a backbone. Genomic regions of HC-Pro and CP (in yellow) amplified from 5-19 were used to replace one or both of the corresponding regions in YK to generate the recombinant viruses YK/519HC, YK/519CP3U and YK/519HCCP3U. **(B)** Resistance evaluation of the PRSV single-virus-resistant transgenic line 18-2-4 (carrying a YK CP-3'UTR transgene as indicated in the upper panel) against YK, 5-19 and the recombinants. Non-transgenic (NT) plants of papaya cultivar Tainung No. 2 were used as a control. Symptoms were recorded 30 days post mechanical inoculation (dpi). **(C)** Resistance evaluation of the PRSV-PLDMV double-resistant-transgenic line 14-1 (carrying a chimeric construct comprised of parts of PLDMV CP and PRSV CP-3'UTR, as indicated above the panel) against YK, 5-19 and the recombinants. Symptoms were recorded 30 dpi.

RNA viruses can be recombined at the cDNA level in the laboratory. Using an infectious, full-length *in vitro* YK cDNA clone as a backbone^27^, which can generate a full-length, infectious YK RNA genome following *in vitro* transcription from a bacteriophage T3 promoter, the recombinant viruses YK/519HC and YK/519CP3U were generated by replacing the HC-Pro or/and CP-3'UTR regions of YK with the corresponding sequences from 5-19 (Fig. 1A).

A total of 30 plants, each from the PRSV single-virus-resistant lines 16-0-1,17-0-1,18-2-4 and 18-0-9^21^, were mechanically inoculated with YK, 5-19 or the recombinant viruses under greenhouse conditions in four independent tests. Two months after inoculation, plants from lines 18-2-4 and 17-0-1 were completely resistant to YK infection, whereas 3 and 13% of the plants from lines 16-0-1 and 18-0-9 were infected with YK, respectively. The recombinant viruses YK/519HC and YK/519CP3U caused symptoms of severe leaf mosaic and distortion in non-transgenic (NT) papaya plants two weeks after inoculation. At two months post inoculation, YK/519HC and YK/519CP3U caused infection rates of 51% and 53%, respectively, in the four transgenic lines (Fig. 1B; Table 1). Similar symptoms of comparable severity were also caused by YK/519HCCP3U in 56% of the transgenic plants, suggesting the absence of an additive effect in overcoming the resistance of CP-transgenic lines, even with the combined presence of both 5-19 HC-Pro and CP genes in the YK backbone (Fig. 1B; Table 1).

All three recombinants were also able to overcome the resistance of PRSV-PLDMV CP transgenic lines^23^ (Fig. 1C), with infection rates as high as 95-100% in the four tested lines 9-5, 10-4, 12-4, and 14-1^23^ at eight weeks after inoculation (Table 2). Our results indicated that 5-19 HC-Pro enables recombinant YK/519HC, which possesses an identical CP-3'UTR to that of the transgene (100% nt identity), to overcome both single- and double-virus resistances in a sequence homology-independent manner. Because the resistance-breaking ability of 5-19 HC-Pro was not enhanced by the presence of the 5-19 CP-3'UTR in recombinant YK/519HCCP3U (Table 1 and 2), we concluded that 5-19 HC-Pro plays a vital role in the breakdown of nucleotide sequence-homology-independent resistance.

Intriguingly, YK/519CP3U also significantly increased the percentage of breakdown, implying a possible contribution from CP-3'UTR. Because the nt identity of 5-19CP/3'UTR to that of YK is much higher than to the exotic strains HA, TH, and MX, which can not overcome single- and double-virus-transgenic resistance^23^,^24^, the breakdown mediated by the recombinant YK/519CP3U is also probably not sequence-homology-dependent. However, we cannot exclude the possibility of homology-dependent breakdown mediated by the heterologous 5-19 CP and 3'UTR in recombinant YK/519CP3U because the two virus strains still show very little nucleotide divergence (95.9% identity for CP and 97.9% for 3'UTR).

### Breakdown of CP-transgenic resistance is due to suppression of the host defence reaction

**Figure 2:**
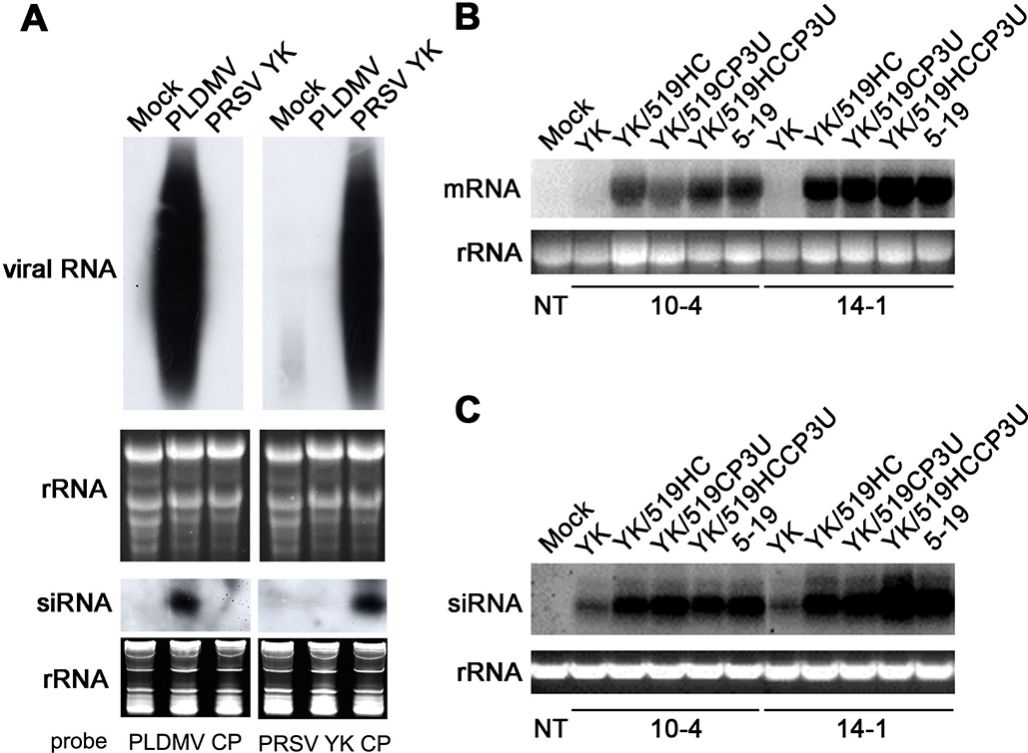
Northern blot detection of the chimeric CP transcript and the corresponding siRNA in plants from double-virus-resistant transgenic papaya lines after challenge inoculation. Plants from transgenic lines 10-4 and 14-1, which carry the PLDMV/PRSV chimeric CP construct, were separately inoculated with PRSV 5-19, YK and the recombinant viruses YK/519HC,YK/519CP3U and YK/519HCCP3U. Total RNAs were extracted at 30 days after virus inoculation and used for hybridisation. **(A)** For non-transgenic plants, the radiolabelled PLDMV CP or PRSV CP probe detected the hybridisation signals of viral RNA and siRNA only in plants infected with the homologous virus. **(B)** The probe for the PLDMV CP coding region detected the chimeric CP transgenic transcript in plants inoculated with PRSV 5-19 or the recombinant viruses but not in plants inoculated with PRSV YK. **(C)** The PLDMV CP probe also detected the corresponding siRNA from plants inoculated separately with PRSV 5-19, YK and the recombinants. Below each northern profile (A, B & C), ethidium bromide-stained total RNA is shown as a loading control.

We investigated the ability of YK, 5-19 and the three recombinant viruses to supress PTGS in double-virus resistant lines (10-4 and 14-1) carrying the chimeric construct (PLDMV CP-PRSV YK CP-3'UTR)^23^. In northern blots, the PLDMV and PRSV CP probes did not cross-hybridise with preparations from non-transgenic plants infected with the heterologous virus (Fig. 2A) due to the low degree of sequence homology (< 60%). Thus, we chose the PLDMV CP probe to detect the chimeric transgenic transcripts to avoid any possible interference from PRSV RNA. PTGS-mediated resistance to YK was clearly observed via the complete silencing of the chimeric transgenic transcript after inoculation with YK (Fig. 2B). However, the probe detected high levels of chimeric transgenic transcript in transgenic plants infected with 5-19 or the three recombinants (Fig. 2B). Our results indicated that 5-19 and the recombinants can infect and establish themselves in the transgenic lines by suppressing the PTGS-mediated degradation of the chimeric transgenic transcripts. This ability to suppress PTGS and compromise the degradation of the transgenic transcripts is apparently due to the different silencing-suppression abilities of 5-19 and the YK recombinants. Once PTGS has been shut off, the chimeric transgenic transcripts and viral RNA are safeguarded from the host gene-silencing machinery.

Because the PLDMV CP probe did not cross-hybridise with the siRNA from PRSV-infected non-transgenic plants (Fig. 2A), the source of the detected siRNA must be the chimeric transgenic transcript. In these same infected transgenic plants, the corresponding 21-22 nt siRNAs were also observed to accumulate to higher levels than in YK-resistant transgenic plants, despite considerable accumulation of the chimeric transcript (Fig. 2C). Potyviral HC-Pro suppresses gene silencing by interfering with the RNA silencing machinery at multiple levels, including (i) reducing siRNA levels by incompletely inhibiting the processing of dsRNA by Dicer^28^,^29^; (ii) preventing mRNA degradation by interfering with the function of the RNA-induced silencing complex (RISC)^29^,^30^, possibly by inhibiting the 3'-methylation of siRNAs, which leads to enhanced oligo-uridylation and subsequent degradation of siRNA^31^,^32^; and (iii) binding and trapping siRNAs to compromise signaling^33^,^34^. Although the binding and trapping of siRNAs by HC-Pro in RDR6-dependent systems can inhibit the maintenance/amplification step and lower the total levels of signalling siRNAs^33^, the sequestered siRNA may accumulate along with the preserved target RNA^33^, as observed in the present study (Fig. 2B&C) and also reported elsewhere^35^.

### PRSV strain 5-19 has a stronger silencing suppressor HC-Pro than other strains

**Figure 3:**
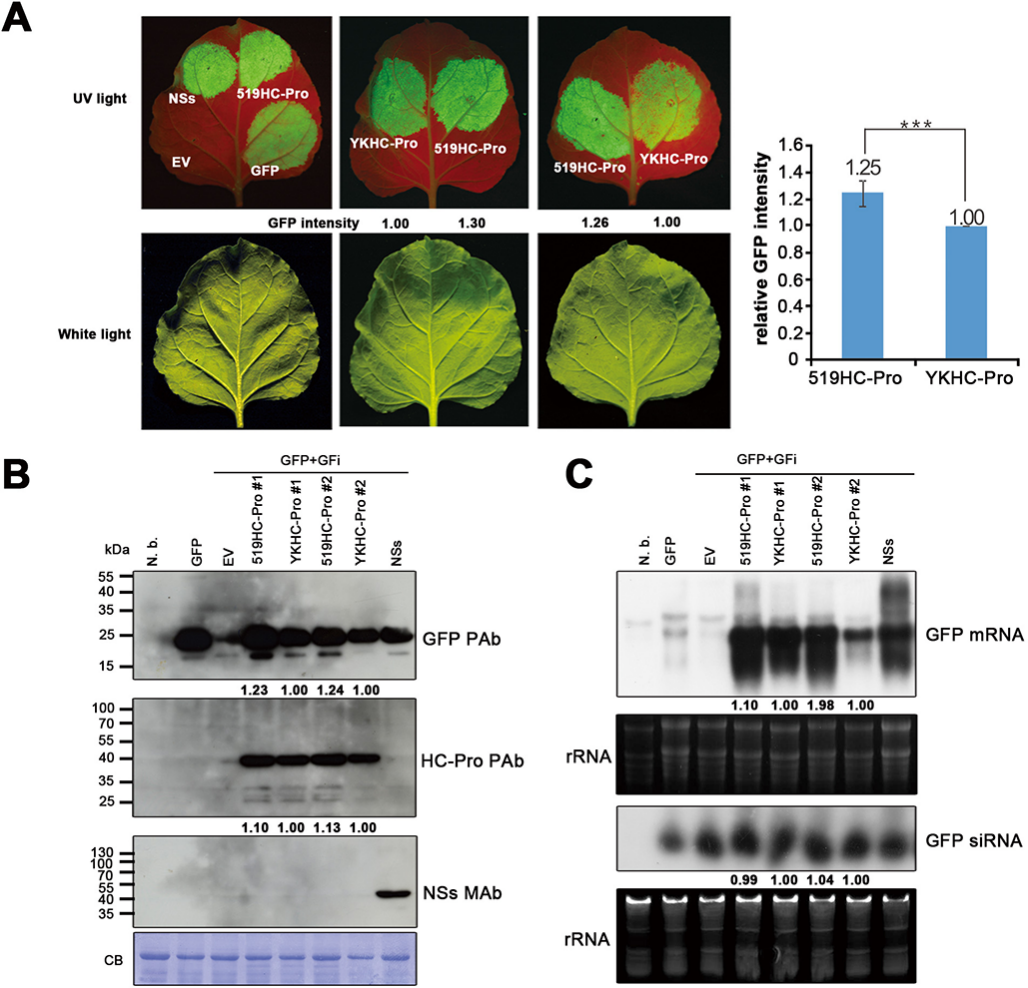
Comparison of the gene silencing suppression capabilities of helper component protease (HC-Pro) from the transgenic-resistance-breaking strain 5-19 and the non-breaking strain YK of *Papaya ringspot virus* (PRSV). The signals from the YK HC-Pro-infiltrated samples were arbitrarily set as 1.0 for each repeat. **(A)** The green fluorescent protein (GFP)-expressing vector pBA-GFP (GFP expressor) was co-infiltrated with the hairpin RNA vector pBA-GFi (silencing inducer), as well as with pBA vector expressing the RNA silencing suppressors PRSV YK HC-Pro, PRSV 5-19 HC-Pro or the *Watermelon silver mottle virus* (WSMoV) NSs protein (positive control), into the leaves of *Nicotiana benthamiana* plants. The empty vector pBA (EV) was used as a non-silencing-suppression control. GFP fluorescence was monitored 5 days post infiltration (dpi). The number indicates the relative GFP intensity of the candidate proteins using YK-HC-Pro as a standard (1.0). Values represent means (n = 6). Value marked with symbols (***) indicate very significant differences (P<0.001). **(B)** Transiently expressed recombinant proteins were detected in infiltrated areas at 5 dpi by chemiluminescent western blotting using 5-19 HC-Pro antiserum, GFP antiserum or WSMoV NSs monoclonal antibody. The lower panel shows Coomassie blue (CB)-stained gels as loading controls. **(C)** The expression of GFP mRNA and the corresponding siRNA in infiltrated areas was monitored by northern hybridisation using a 32P-radiolabelled GFP probe. Ethidium bromide-staining for total RNA is shown under each panel as a loading control.

The gene-silencing-suppression capabilities of 5-19 in comparison with that of YK were compared via *Agrobacterium tumefaciens*-mediated transient expression of their respective HC-Pro constructs in the leaf tissues of *Nicotiana benthamiana* plants that were co-infiltrated with a translatable green fluorescent protein (GFP) construct (reporter) and a GFP hairpin construct (PTGS inducer), as described for *Tobacco etch virus* (TEV) P1/HC-Pro^36^. UV irradiation of the *Agrobacterium*-infiltrated leaves 5 days after infiltration showed that the fluorescence intensity of 5-19 HC-Pro-infiltrated areas was 1.25-fold higher (mean of six repeats) than that of YK HC-Pro-infiltrated areas (Fig. 3A), indicating significantly stronger gene silencing suppression by 5-19 HC-Pro compared with YK HC-Pro (Fig. 3A). Since HC-Pro is the weapon of the virus to counteract the defensive RNA silencing from the host, if a virus mutant (strain) can resist RNA silencing, its titers would increase immediately and subsequently amplify the silencing suppression effect. This suggests that a slight difference would affect the consequence of the fight between the host and the invading virus dramatically. We strongly believe that a 25% increase in silencing suppression would significantly result in different biological phenotype as reflected by the results of this study, especially the virus recombination assays (Fig. 1). Similarly, western and northern analyses revealed higher levels of GFP protein (1.24-fold), HC-Pro protein (1.12-fold) (Fig. 3B) and GFP mRNA (1.54-fold) (Fig. 3C) in 5-19 HC-Pro-infiltrated areas compared with YK HC-Pro-infiltrated areas. The enhanced preservation of GFP transcript might be caused by stronger suppression capability or higher protein stability of 5-19 HC-Pro (Fig. 3B). This experiment was repeated two more times and similar results were noticed. Moreover, GFP siRNAs were detected (Fig. 3C) in 5-19 and YKHC-Pro-infiltrated areas, implying that the sequestration of siRNA by HC-Pro is the principal mechanism of PTGS suppression.

### Slight modifications in HC-Pro may alter its silencing suppression ability

HC-Pro of PRSV 5-19, which shares 96.8/98.7% nt/aa identity (Table S1) with PRSV YK, shows 44 nt and 6 aa variations (YK→5-19): one (V_25_→A) at the N-terminal region and five (R_103_→K, V_118_→I, G_124_→E, G_152_→E, and I_161_→V) in the central region (Fig. S1A, B), with the latter being responsible for viral replication and PTGS suppression^37^ (Fig. S1C). HC-Pro sequences from diverse strains to which the YK CP-3'UTR-transgenic plants were resistant share 95-97% aa identity with 5-19 HC-Pro (Table S1). Potyviral HC-Pro is a multifunctional protein with different domains that fulfil its multiple roles^38^, and slight changes in these different functional domains may have a concerted effect on silencing suppression. Our results revealed that there was no correlation between particular sequence differences and the ability to break CP-transgenic resistance.

### An approach for solving the problem of CP sequence-homology-independent transgenic resistance breakdown

**Figure 4:**
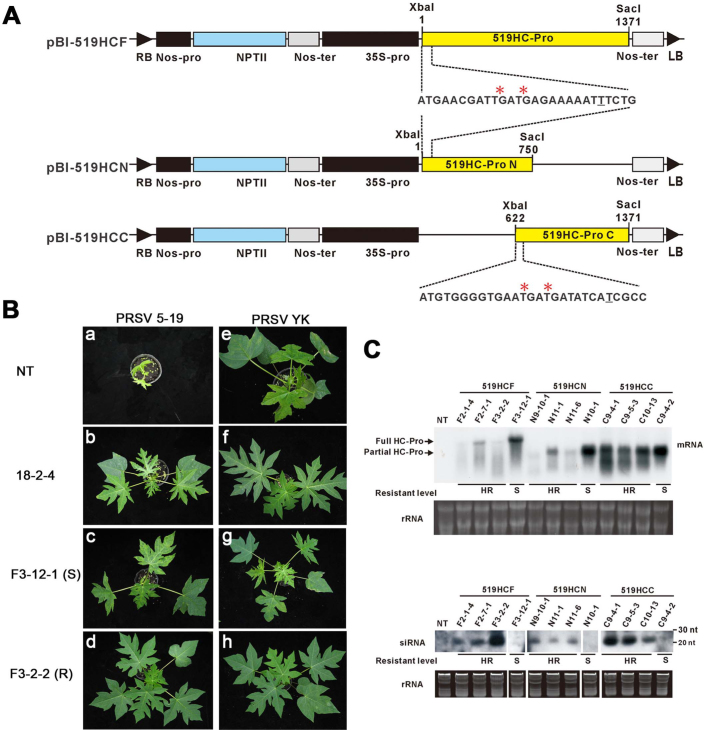
Generation of transgenic papaya resistant to the CP-transgenic-resistance-breaking strain 5-19 of *Papaya ring spot virus* (PRSV) by gene silencing targeting at viral helper component protease (HC-Pro) coding region. The organisations of T-DNA engineered to contain the full-length, N-terminal or C-terminal 5-19 HC-Pro coding sequence in the transformation vectors pBI-519HCF, pBI-519HCN and pBI519HCC, respectively, are shown. The constructs were controlled by a CaMV 35S promoter and a *nos* terminator and are untranslatable due to the presence of two termination codons (asterisks) and an inserted T residue (underlined) that causes a frame shift. The T-DNAs also carry a *npt*-II marker gene under the control of a *nos* promoter and a *nos* terminator. **(B)** Plants of the non-transgenic papaya cultivar Tainung No. 2 (NT), the PRSV YK CP-3'UTR transgenic single-virus-resistant line 18-2-4, and the PRSV 5-19 HC-Pro transgenic lines F3-12-1 (susceptible) and F3-2-2 (resistant) were inoculated with PRSV 5-19 or YK, and symptoms were recorded 28 days post inoculation. **(C)** Northern blot detection of HC-Pro transgenic transcripts and the corresponding siRNAs from 5-19-highly resistant (HR) or susceptible (S) PRSV 5-19 HC-Pro transgenic lines. Ethidium bromide-stained total RNAs are shown under the mRNA (upper) and siRNA (lower) hybridisation panels as loading controls. For northern blotting hybridisation, all the RNA samples were electrophoretically resolved and blotted under uniform conditions, and all the blots were hybridised in a single reaction.

Three transformation vectors, pBI-519HCF, pBI-519HCN and pBI-519HCC, carrying untranslatable full-length (nt 1-1371), N-terminal (nt 1-750) and C-terminal (nt 622-1371) 5-19 HC-Pro, respectively, were constructed. Each construct carries two engineered termination-codons, along with an inserted T nucleotide that causes frame shift, and is controlled by the CaMV 35S promoter and *nos* terminator (Fig. 4A). Somatic embryos derived from *in vitro* adventitious roots^39^ of papaya cultivar Sunrise were transformed using *A. tumefaciens* harbouring the individual vectors, and 60 transgenic lines were established (Table S2).

Micropropagated 5-19 HC-Pro transgenic plants were evaluated for their resistance to PRSV by mechanical inoculation with 5-19 or YK. Non-transgenic (NT) plants and plants from YK-CP-3'UTR transgenic line 18-2-4^21^ were used as controls. Two weeks after inoculation in NT plants, 5-19 induced stunting and severe mosaic symptoms (Fig. 4B-a), which subsequently deteriorated into lethal wilting; mosaic symptoms were also observed in 18-2-4 plants (Fig. 4B-b). Thirty-one 5-19 HC-Pro transgenic lines were susceptible to 5-19 (Table S2; Fig. 4B-c), thirty of which were also susceptible to YK (Table S2), as defined by no delays in the development of symptoms. Of the twenty-nine 5-19-resistant transgenic lines, 10 were weakly resistant (WR) (Table S2), as defined by a delay in the development of symptoms, and 19 were highly resistant (HR) to 5-19 (Table S2; Fig. 4B-d), as defined by symptomless appearance and negative ELISA results 8 wk after inoculation. PRSV YK induced severe mosaic symptoms in NT plants (Fig. 4B-e). As reported earlier^21^, the plants from the 18-2-4 line were resistant to YK (Fig. 4B-f). The susceptible lines developed severe non-lethal vein-clearing and leaf-mosaic symptoms after inoculation with 5-19 or YK (Fig. 4B-c & g), whereas all 19 HR lines were also highly resistant to YK (Table S2; Fig. 4B-h).

A significantly higher percentage of HR lines (46.0%) was found among the full-length HC-Pro lines than among lines carrying the N-terminal 519HCN construct (29.0%) or the C-terminal 519HCC construct (21.4%) (Table S2). Southern hybridisation analysis of sixteen of lines that were highly resistant to both YK and 5-19 indicated the presence of 1-6 HC-Pro transgene inserts (Fig. S2). We also obtained 12 lines of 5-19 CP transgenic papaya, however, they did not show significant degrees of resistance to both PRSV 5-19 and YK strains (data not shown). Because high percentages of transgenic lines with different constructs of 5-19 HC-Pro (average 31.6%, Table S2) resistant to 5-19 were obtained, we did not proceed further experiments to obtain more 519-CP lines for comparison.

Four HR lines (F2-1-4, F2-7-1, F3-2-2, and N11-1) with single insertions of the untranslatable 5-19 HC-Pro construct were further analysed for their resistance to different geographical PRSV strains. Line F2-7-1 was susceptible to the exotic PRSV strains HA, TH and MX. Lines F2-1-4 and N11-1 showed delayed symptoms to HA and TH but were susceptible to MX (Table S3). However, line F3-2-2 showed complete resistance to all the exotic strains tested and was thus regarded as an extremely HR line.

Of the nine HR lines and three S lines (three HR and one S lines for each full-length, N-half, and C-half untranslatable HC-Pro) analysed by northern hybridisation, five HR lines (F2-7-1, N11-1, C9-4-1, C9-5-3, C10-13) accumulated significantly lower levels of transgenic transcripts than the S lines. However, transgenic transcripts were not detectable in the other four HR lines (Fig. 4C). In addition, siRNAs were detected in all the nine HR lines but not in any of the S lines (Fig. 4C). The highest level of siRNA was detected in the single-insert full-length HC-Pro transgenic line F3-2-2 that conferred broad-spectrum resistance to 5-19 and all other PRSV strains (Table S3). Our results demonstrated that the transgenic resistance to the virulent virus 5-19 and the other PRSV strains is based on the targeting of the gene-silencing suppressor HC-Pro by PTGS. However, HC-Pro resistance is not merely dependent on sequence identity but also on its function as a suppressor.

### Segregation analysis of the *npt*II gene in R_1_ progeny

The segregation ratios of the *npt*II gene in the R_1_ progeny from the 2 HR lines (F2-1-4 and F3-2-2) were 3:1, as calculated by the ratios of geminating seedling resistant or sentive on the selection medium containing kanamycin (Table S4), verifying that the *npt*II gene was integrated into the chromosome at a single locus, as revealed by Southern blotting (Fig. S2) . When these kanamycin-resistant plants were mechanically challenged with PRSV 5-19, all of them were resistant to viral infection. Thus, our results further indicated that the transgenic resistance to 5-19 is linked with kanamycin resistance and undergoes nuclear inheritance as a dominant trait.

## Discussion

The severity of viruses can be increased by a few mutations in gene silencing suppressors^34^. As demonstrated by the present investigation, such mutant viruses can overcome PTGS-mediated transgenic resistance independent of transgene sequence homology. Although transgenic papaya lines grown in Hawaii are not resistant to PRSV strains from other geographic areas, no breakdown in the transgenic resistance to PRSV strains indigenous to Hawaii has been observed^3^. The geographical isolation of Hawaii restricts the diversity of PRSV strains and contributes to the stable and continuous effectiveness of PRSV CP-transgenic papaya for more than a decade^18^. However, the emergence of more virulent virus strains through the selection mechanism described in this study is expected to prevail in areas where more diverse PRSV strains exist.

Influenced by European trends, the government of Taiwan has yet to approve any of the transgenic crops cultivated on the island. Nevertheless, field tests of CP-transgenic papaya lines under isolated conditions have been strongly supported by the government over the past two decades. During more than 10 years of regulated field trials, the complexity of the viruses that infect CP-transgenic papaya lines has helped advance the development of single-virus^21^, double-virus^22^,^23^, and virulent virus (this study) resistance to PRSV, PRSV/PLDMV, and virulent PRSV strains, respectively. The lethal wilting type PRSV 5-19 with a stronger HC-Pro described herein infects the YK-CP-3'UTR-transgenic papaya plants as a non-lethal mosaic virus. Here, we demonstrate that the virulent strain 5-19 exhibits a strong ability to suppress gene silencing and overcome CP-transgenic resistance, which allows it to establish in CP-transgenic plants and cause non-lethal mosaic symptoms. The emergence and accumulation of such virulent strains in CP-transgenic plants poses a serious risk that may completely wipe out CP-transgenic and non-transgenic papaya plantations and also threatens other cucurbitaceous crops that can be infected by PRSV. Our results further indicate that the breakdown of CP-transgenic resistance by virulent strains, such as PRSV 5-19, is independent of the genetic diversity of PRSV CPs. Thus, regardless of investigations into the diversity of CPs to develop CP-transgenic resistance against different PRSV strains present in a particular region, CP-transgenic plants could still facilitate the emergence of PRSV mutants or variants with stronger RNA silencing suppression from amongst the natural virus population. This complexity emphasises the importance of monitoring the possible emergence of more virulent virus strains during the cultivation of CP-transgenic crops.

Our study also demonstrated that untranslatable constructs targeting the gene silencing suppressor HC-Pro can disarm viral protections against host defences and minimise the potential emergence of more virulent virus strains that can overcome CP-transgenic resistance. Currently, we are pyramiding single-virus, double-virus, and virulent virus transgenic resistances in papaya to develop a hybrid variety with broad-spectrum resistance for global application and reduced risk for the emergence of more virulent virus strains during cultivation.

## Methods

### Virus isolates

*Papaya ringspot virus* (PRSV) isolates YK^40^, 5-19^24^, HA^41^, TH and MX^21^ and *Papaya leaf-distortion mosaic virus* (PLDMV) isolate P-TW-WF^22^ were used in this study. PRSV YK, a typical mosaic type strain collected from southern Taiwan, was previously characterised and its genome sequenced^40^. PRSV 5-19^24^ and PLDMV P-TW-WF^22^, both severely infecting transgenic papaya (*Carica papaya* L. cultivar Tainung No. 2) lines carrying the PRSV YK CP coding region along with the contiguous PRSV YK 3'-UTR^21^, were collected from central Taiwan during field trials^42^. The geographically distant PRSV strains HA^41^, TH and MX^21^, which originate from Hawaii, Thailand and Mexico, respectively, were used to evaluate the broad-spectrum resistance of the transgenic papaya lines. All virus isolates were maintained in papaya (*Carica papaya* L. cv. Tainung No. 2) plants.

### Construction of viral recombinants between PRSV YK and 5-19

The *in vitro* infectious PRSV YK cDNA clone pT3PYKF1, driven by the bacteriophage T3 promoter, was constructed in our previous study^27^. A cDNA fragment corresponding to the HC-Pro gene of PRSV 5-19 was amplified by RT-PCR from total RNA isolated from 5-19-infected non-transgenic papaya plants (Ultraspect M RNA isolation system, Biotecx laboratories, Houston, TX). First-strand (fs) cDNA was synthesised using the primer Myk3139 (5'-GGCTTGTAAATGACGCGTATTAATTGATGC-3') and M-MLV reverse transcriptase (Promega, Madison, WI, USA). A DNA fragment containing the HC-Pro region of PRSV 5-19 RNA^43^ (nts 1640 to 3156) was PCR amplified from fs-cDNA using the primers Pyk1646 (5'-TTCATCACGCGTGGGCGTTACGCA-3') and Myk3139. The *in vitro* infectious recombinant PRSV YK cDNA clone pT3YK/519HC, in which the PRSV YK HC-Pro region was replaced with the corresponding region from PRSV 5-19, was constructed by ligating the *Mlu*I-digested HC-Pro gene region of 5-19 into *Mlu*I-digested pT3YK (Fig. 1A).

Because the N-terminal region of the CP coding region of PRSV contained a poly(A) tract, the cDNA corresponding to the PRSV 5-19 CP coding region was amplified as two partial fragments. The N-terminal part was amplified using the primers YK905 (5'-GCAGGGCCCCATATGTGTCT-3', *Apa*I site underlined) and Mo1008 (5'-GTGCATGTCTCTGTTGACAT-3') and cloned into a PCR-TOPO vector (Invitrogen, San Diego, CA, USA) to generate p519 (905-1008)/TA. The C-terminal part, along with the contiguous 3'-UTR, was amplified using the primers TL (5'-CTAGATATGCTTTCG-3') and oligo-dT_(18)_NS (5'-AATTGAGCTCGCGGCCGCTTTTTTTTTTTTTTTTTT-3', *Not*I site underlined) and cloned into a PCR-TOPO vector to generate p519(TL-oligodt)/TA. The *Apa*I-*Pst*I restriction fragment from p519(905-1008)/TA and the *Pst*I-*Not*I restriction fragment from p519(TL-oligodt)/TA were ligated and cloned together into pBluescriptII SK+ to generate p519CP/SK+ carrying the complete CP coding region and contiguous 3'-UTR (CP-3'-UTR) of PRSV 5-19. The *in vitro* infectious PRSV YK recombinant cDNA clone pT3YK/519CP3U, in which the PRSV YK CP-3'-UTR was replaced with that from PRSV 5-19, was constructed by replacing the *Apa*I-*Not*I region of pT3PYKF1 with that from p519CP/SK+ (Fig. 1A). Another *in vitro* infectious PRSV YK recombinant cDNA clone, pT3YK/519HCCP3U, in which both PRSV YK HC-Pro and CP-3'UTR were replaced with the corresponding regions from PRSV 5-19, was also constructed (Fig. 1A).

### Confirmation of recombinant viruses in infected plants

*Not*I-linearised constructs for the wild-type and recombinant viruses were transcribed *in vitro*, and the products were mechanically introduced into papaya seedlings (cv. Tainung No. 2) as described previously^44^. The inoculated plants were maintained in a temperature-controlled (25 ± 3°C) greenhouse to observe symptom development.

Appropriate cDNAs amplified by RT-PCR using RNA extracted from virus-infected papaya plants were analysed by restriction-enzyme digestion and sequencing to confirm the identities of the recombinant viruses. cDNA containing the HC-Pro coding region was amplified using the PYK1646/MYK3139 primer pair and analysed by *Xho*I digestion to specifically release the fragment corresponding to the 5-19 HC-Pro gene. cDNA containing the CP coding region along with the 3'-UTR was amplified by RT-PCR using the YK905/YK10326 primer pair and then sequenced to verify its origin from PRSV 5-19.

### Infectivity of recombinant viruses in transgenic papaya lines

Infectivity assays were performed with (i) the single-virus-resistant YK CP-3'UTR transgenic papaya lines 18-2-4, 18-0-9, 17-0-1 and 16-0-1^21^, which carry a transgene corresponding to the PRSV YK CP coding sequence with the contiguous 3' UTR (CP-3'UTR), and (ii) the double-virus-resistant PY16 transgenic papaya lines 9-5, 10-4, 12-4 and 14-1^23^, which carry a chimeric transgene comprising parts of both PLDMV CP and PRSV YK CP-3'UTR. Both the single-virus-resistance to PRSV^21^ and the double-virus-resistance to PLDMV and PRSV^23^ are based on post transcriptional gene silencing (PTGS).

All transgenic lines were evaluated against the recombinant viruses YK/519HC, YK/519CP3U and YK/519HCCP3U (Fig. 1A), which were derived from the pT3YK/519HC, pT3YK/519CP3U and pT3YK/519HCCP3U constructs, respectively, by mechanical inoculation, with YK and 519 serving as controls. Inocula of the challenge viruses were prepared by extracting leaf tissues from virus-infected papaya plants (cv. Tainung No. 2) with 20 volumes (w/v) of 0.01 M phosphate buffer (pH 7.0) at 15 days after inoculation with the individual viruses. Plants from the YK CP-3'UTR transgenic lines were inoculated on two fully expanded upper leaves at the 10-cm stage. The infection rates from four independent tests, with a total of 30 plants/ line, were statistically analysed using Duncan’s multiple range test. Plants from the double-virus resistant PY16 lines were similarly inoculated at the 15-cm stage, and two experiments were performed, with a total of 20 plants/line. All inoculated plants were kept in a temperature-controlled (25 ± 3°C) greenhouse for two months after inoculation to evaluate their resistance.

### Indirect enzyme-linked immunosorbent assay

Indirect ELISA was performed as described previously^45^, with modifications. Leaf extracts diluted (1:100) in 50 mM sodium carbonate buffer (pH 9.6) with 0.01% sodium azide were used to coat the wells of a polystyrene microtiter plate. PRSV CP antiserum^46^ diluted 2000-fold in conjugate buffer^46^ was added (200 μl/well) and incubated at 37°C for 1 hr. After the wells were washed the three times, 200 μl of alkaline phosphatase-conjugated goat anti-rabbit immunoglobulin (KPL, Inc., Gaithersburg, MD, USA), diluted 5000-fold in conjugate buffer, was added to each well. After additional washing, 100 μl of 1 mg/ml *p*-nitrophenyl phosphate (Sigma-Aldrich Corporation, St. Louis, MO, USA) in substrate buffer (100 mM diethanolamine, pH 9.6) was added to each well. The absorbance was measured at 405 nm on a Rainbow microplate reader (SLT Lab Instruments, Winosski, VT, USA) 30 min after the addition of enzyme substrate. Transgenic plants that exhibited two-fold or higher absorbance compared with the negative control were classified as ELISA positive.

### Preparation of 5-19 HC-Pro antiserum

Amorphous inclusions of PRSV 5-19 were purified from infected *Cucumis metuliferus* (Naud.) Mey. (Acc. 2459) using the previously described differential centrifugation method^47^. The HC-Pro protein was further dissociated and then purified by preparative gel electrophoresis as described^46^,^48^. BALB/c mice were immunised by intraperitoneal injection of 50 µg of purified HC-Pro protein once a week for three weeks. The antiserum was collected to detect HC-Pro from PRSV 5-19 and YK in the agro-infiltration assay.

### Analysis of the gene-silencing-suppression ability of HC-Pros of 5-19 and YK by agro-infiltration

*Nicotiana benthamiana.* Domin plants at the 10-cm stage were used for agro-infiltration. The HC-Pro coding regions of PRSV 5-19 and YK were amplified using the primers HC-Pro-F (5'- CACCATGAACGATATTGCTGAAAAATTC-3') and HC-Pro-R (5'-GCTCACTAGTTTTAACCGACAATGTA-3') and inserted into the pBA-DC-HA vector^49^ to generate pBA-519HC and pBA-YKHC, respectively.

Suspensions of *Agrobacterium tumefaciens* ABI carrying pBA-GFP (a GFP expressor, driven constitutively by a 35S promoter, prepared in our laboratory) and pBA-GFi (a 2/3 GFP ORF construct with an inverted repeat to induce silencing, provided by Dr. Shih-Shun Lin, National Taiwan University) were mixed (ratio 2: 1: 2) with the individual pBA-519HC, pBA-YKHC and pBA-NSs vectors containing 5-19 HC-Pro, YK HC-Pro and the NSs coding region (silencing suppressor positive control) of *Watermelon silver mottle virus* (WSMoV)^48^, respectively, and then injected into the lower side of leaves using a syringe without a needle, and the infiltrated plants were kept in a temperature controlled (25 ± 3°C) greenhouse. The empty vector pBA^49^ was used as a negative control. GFP fluorescence was monitored at 5 dpi using a hand-held UV light B-100AP (UVP, CA, Upland, USA) and photographed using a digital camera (D7000, Nikon, Tokyo, Japan) with a Cokin P series filter (Cokin, 84 mm, 524 nm, Rungis, French). To minimize the variation in leaf age, size and position, half leaf assay was performed (n = 6). The GFP fluorescence signals were quantified by Kodak 1D image analysis software (Eastman Kodak, Rochester, NY). The density of GFP fluorescence in YK HC-Pro infiltrated leaves was arbitrarily set as 1:00 to relatively quantify that in the same half leaves infiltrated with 5-19 HC-Pro. The transient expression of recombinant proteins was monitored at 5 dpi via chemiluminescent western blotting (Amersham, Bucks, U.K.) using 5-19 HC-Pro antiserum (prepared as described above), GFP antiserum^50^, or WSMoV NSs MAb^48^ as the primary antibody and horseradish peroxidase-conjugated goat anti-rabbit immunoglobulin (Amersham, Bucks, U.K.) or horseradish peroxidase-conjugated goat anti-mouse immunoglobulin (Amersham, Bucks, U.K.) as the secondary antibody.

The protein signals were quantified by Kodak 1D image analysis software (Eastman Kodak, Rochester, NY).

### Northern hybridisation for mRNA/siRNA detection

For the detection of particular transcripts, total RNA was extracted from young leaves of transgenic and non-transgenic papaya plants (cv. Tainung No. 2) using the ULTRASPEC^TM ^RNA isolation system (Biotecx Laboratories Inc., Houston,TX,USA). Fifteen micrograms of total RNA was separated on a 1.2% agarose gel with formaldehyde, transferred to a Gene Screen Plus nylon membrane (Dupont Co., Boston, MA) and hybridised with α-32P-dATP-labeled probes specific to the coding sequences from PLDMV CP, PRSV YK CP, GFP or PRSV 5-19 HC-Pro. The probes were prepared using the Primer-It II random primer labelling kit (Stratagene, La Jolla, CA) from 25 ng DNA amplified from the corresponding constructs using appropriate primers. Prehybridisation, hybridisation, washing, and autoradiography were carried out as described^23^.

For the detection of the siRNA derived from the transgenic constructs, thirty micrograms of total RNA was resolved on a 15% polyacrylamide/1×TBE (8.9 mM Tris-HCl, 8.9 mM boric acid, 20 mM EDTA)/8 M urea gel and transblotted to a Hybond-N+ membrane (Amersham Phamacia Biotech, Bucks, UK). For hybridisation, α-32P-labeled probes were prepared as described above. Hybridisation was carried out in ULTRAHyb-Oligo solution (Ambion Inc., Austin, TX), and signals were detected by autoradiography. The Dynamarker® Prestain Marker (BioDynamics Laboratory Inc., Tokyo, Japan) for small RNA was used as the molecular weight standard. The mRNA/siRNA signals were quantified by Kodak 1D image analysis software (Eastman Kodak, Rochester, NY).

### Cloning and sequencing of PRSV TH and MX HC-Pro coding sequences

Total RNA was isolated from papaya leaves infected with PRSV TH or PRSV MX 3 weeks after inoculation. The primer MYK3234 (5'-GTTAAAAGTACGCTTGGTGACATC-3'), which corresponds to nucleotide positions 3211-3235 in the PRSV YK RNA sequence, was used for first-strand cDNA synthesis by reverse transcription (RT). The forward primer PPYK1607 (5'-GCTGACAGAATAGGTCGATA-3'), which corresponds to nucleotide positions 1607-1626 in the PRSV YK RNA sequence, was used with the reverse primer MYK3234 to PCR amplify the HC-Pro gene from each virus . RT-PCR-amplified PRSV TH and MX HC-Pro fragments were cloned into pCR-TOPO (Invitrogen, San Diego, CA, USA) and sequenced.

The nucleotide and amino acid sequences of HC-Pro from PRSV TH and MX were compared with those from 5-19, YK^40^ and HA^43^ using PC/GENE 6.85 software (IntelliGenetics, Inc., Mountain View**,** CA, USA). Multiple sequence alignments were constructed using the PILEUP program of the GCG package, version 9.0 (Genetics Computer Group, Madison, WI, USA).

### Construction of untranslatable PRSV 5-19 HC-Pro constructs

To construct untranslatable PRSV 5-19 HC-Pro coding sequence in binary vector, the forward primer 519HCStopA (5'-CGCATGAACATGCTCTAGATGAACGAT**TGATGA**GAAAAAT***T***TCTG-3'), which contains two stop codons (bold), an *Xba*I site (underlined) and an extra thymidine (T) nucleotide (italicised) inserted to create frame-shift, and the reverse primer 519HCSacIB (5'-GTGGTTGGATCAAAGAGCTCACCGACAATGTAGTGTTTCATTTC-3', *Sac*I site underlined) were used to amplify the frame-shifted full-length PRSV 5-19 HC-Pro (1371 bp) coding sequence containing two upstream stop codons, using pT3YK/519HC as the template. The amplified fragment was digested with *Xba*I/*Sac*I and ligated into *Xba*I/*Sac*I-digested pBI121 to generate the untranslatable construct pBI-519HCF.

In addition, the primers 519HCStopA and 519HCSacA (5'-TTTGTGGAGAATATGAGCTCACCAATGGCAACCTTTCGAATG-3', *Sac*I site underlined) were used to amplify the N-terminal fragment (750 bp) of the PRSV 5-19 HC-Pro gene. The amplified product was digested with *Xba*I/*Sac*I and ligated into *Xba*I/*Sac*I-digested pBI121 to generate the untranslatable construct pBI-519HCN.

The primers 519HCStopB (5'-GACAGAAATGGGCTCTAGATGTGGGGT GAA**TGATGA**TATCA***T***CGCCAAAAG-3'), containing two stop codons (bold), an *Xba*I site (underlined) and an inserted extra nucleotide (italicised) for frame shifting, and 519HCSacB were used to amplify the C-terminal fragment (750 bp) of the PRSV 5-19 HC-Pro gene. The amplified product was digested with *Xba*I/*Sac*I and ligated into *Xba*I/*Sac*I-digested pBI121 to generate the untranslatable construct pBI-519HCC. All three untranslatable constructs were transferred into *A. tumefaciens* LBA4404 by electroporation using a Gene Pulser (BioRad, Hercules, CA, USA) as previously described^51^.

### Generation of HC-Pro transgenic papaya lines

Papaya transformation and regeneration were carried out as described previously^39^, with the required modifications. Embryogenic tissues derived from the adventitious roots of the PRSV-susceptible papaya cultivar Sunrise wounded in liquid-phase by carborundum were transformed using *A. tumefaciens* carrying pBI-519HCF, pBI-519HCN or pBI-519HCC. After selection on kanamycin (100 mg/l) medium for 3 months and subsequent growth without kanamycin for one month, the mature somatic embryos were germinated on MSNB shoot medium [MS medium with 0.02 mg/l α-naphthaleneacetic acid (NAA) and 0.2 mg/l BAP] containing 100 mg/l kanamycin for shoot development. Shoot regeneration, micropropogation and acclimatisation were carried out as described previously^20^. The micropropagated R_0_ plants from individual putative transformed shoots were established in vermiculite soil under temperature-controlled (25 ± 3°C) greenhouse conditions^20^ and used for further evaluation. The presence of the transgene in the putative transgenic lines was checked by PCR using the primer pairs 519HCStopA/519HCSacB, 519HCStopA/519HCSacA or 519HCStopB/519HCSacB. The *npt*II-specific primers NA and NB^23^ were also used for PCR analysis to confirm the presence of the selection marker.

### Evaluation of HC-Pro transgenic lines for resistance to 5-19 and YK

The viral resistance of the putative HC-Pro transgenic lines was evaluated by mechanical inoculation with PRSV 5-19^24^ or PRSV YK^40^ using sixty micropropagated R_0 _plants (5 plants/line) at approximately the 15-cm stage. The first two fully expanded leaves were dusted with 600-mesh carborundum and rubbed with 200 μl of inoculum that had been prepared from papaya leaf tissues 3 weeks after inoculation with PRSV 5-19 or PRSV YK (1:20 w/v in 0.01 M potassium phosphate buffer, pH 7.0). Non-transgenic papaya plants (cv. Sunrise) and YK-CP3'-UTR transgenic papaya line 18-2-4^21^ were used as controls. Plants were kept in a temperature-controlled greenhouse (25 ± 3°C), and symptom development was monitored for 7 weeks.

### Southern hybridisation

The DNeasy Plant Mini kit (Qiagen Inc., Valencia, CA, USA) was used to extract genomic DNA from HC-Pro transgenic papaya plants. Thirty micrograms of genomic DNA was digested with *Ase*I (two *Ase*I sites are located at nt 4764 and 4823 in pBI121, prior to the 35S promoter) and the electrophoretically resolved profile (0.8% agarose gel) was transferred to a Gene Screen Plus nylon membrane (Dupont Co., Boston, MA, USA). The Primer-It II random primer labelling kit (Stratagene, La Jolla, CA, USA) was used to prepare the α-32P-dATP-labeled HC-Pro probe from 25 ng of DNA (1371 bp) amplified from pBI-519HCF using the primer pair 519HCStopA/519HCSacB. Southern hybridisation and autoradiography were performed as described previously^23^.

### Resistance of HC-Pro lines to different geographic PRSV strains

Plants from four highly resistant transgenic lines that contained a single transgene insert, as identified by Southern blotting, were tested against geographically distinct PRSV strains from Hawaii (HA)^43^, Thailand (TH)^21^, and Mexico (MX)^21^ by mechanical inoculation, as described above.

### Analysis of transgene segregation in R_1_ progeny

R_0_ plants from two highly resistant 5-19 HC-Pro transgenic papaya lines, F2-1-4 and F3-2-2, were self-crossed under greenhouse conditions, and the R_1_ premature seeds were collected at 90 days after pollination to analyse the inheritance of the transgene. To analyse the pattern of inheritance of the *npt*II gene, the R_1_ premature seeds of F3-2-2 were cultured on MSNB medium containing 100 mg/l kanamycin for two weeks, until plantlets developed. The kanamycin-resistant R_1_ progeny were further established and challenged by mechanical inoculation with PRSV 5-19, as described above, to ascertain the inheritance of the transgenic resistance.

## Supplementary Material

Supplementary Information Supplementary Information

## Figures and Tables

**Table 1:** Reactions of single-virus-resistant transgenic papaya lines carrying CP 3'UTR from *Papaya ringspot virus* (PRSV) YK after inoculation with PRSV YK, virulent strain 5-19, or individual recombinants carrying heterologous HC-Pro (YK/519HC), CP 3'UTR (YK/519CP3U), or both (YK/519HCCP3U) from 5-19.

Virus	YK CP-3'UTR transgenic line					Non-transgenic Tainung No. 2	
	18-2-4	17-0-1	16-0-1	18-0-9	Mean		
YK	0	0	3	13	4^a^	100
5-19	63	53	68	71	64^b^	100
YK/519HC	54	47	57	46	51^b^	100
YK/519CP3U	50	35	62	63	53^b^	100
YK/519HCCP3U	75	47	63	39	56^b^	100

Numbers indicate the percentages of plants showing symptoms two months after inoculation. Four independent tests, comprising a total of 30 plants from each line, were conducted for this assay. Adjacent mean values with the same superscript are not significantly different (P<0.05) based on Duncan’s multiple range test.

**Table 2:** Reactions of double-virus-resistant transgenic papaya PY16 lines carrying an untranslatable construct containing the N-terminal part of the coat protein (CP) coding region of *Papaya leaf-distortion mosaic virus* and the C-terminal part of the CP coding region with the entire 3' untranslated region (3'UTR) of *Papaya ringspot virus* after inoculation with PRSV YK, virulent strain 5-19, or individual recombinants carrying heterologous HC-Pro (YK/519HC), CP coding region plus 3'UTR (YK/519CP3U), or both (YK/519HCCP3U) from 5-19.

Virus	PY16 - transgenic line								Non-transgenic Tainung No. 2							
	9-5		10-4		12-4		14-1									
	3^a^	8	3	8	3	8	3	8	3	8						
YK	0^b^	0	0	0	0	0	0	0	100	100	
5-19	100	100	90	100	100	100	67	100	100	100	
YK/519HC	100	100	89	100	100	100	67	100	100	100	
YK/519CP3U	100	100	95	100	100	100	100	100	100	100	
YK/519HCCP3U	100	100	84	100	100	100	78	95	100	100	

^a^Numbers indicate the weeks after inoculation. ^b^Numbers indicate the percentages of plants showing symptoms at three or eight weeks after inoculation. Two experiments were conducted, comprising a total of 20 plants in each line.
