# Supplementary material for: Nucleotide Sequence-Homology-Independent Breakdown of Transgenic Resistance by More Virulent Virus Strains and a Potential Solution
Source: Sci Rep. 2015 Apr 27;5:9804. doi: 10.1038/srep09804 (PMC5386206; doi:10.1038/srep09804)
Supplement: Supplementary Information [file srep09804-s1.doc]

**Supplementary data for**

**Nucleotide Sequence-Homology-Independent Breakdown of Transgenic Resistance by More Virulent Virus Strains and a Potential Solution**

Yi-Jung Kung^1,2^, Bang-Jau You^1,3^,Joseph A. J. Raja^1,2^, Kuan-Chun Chen^1^, Chiung-Huei Huang^1^, Huey-Jiunn Bau^1^, Ching-Fu Yang^1,4^, Chung-Hao Huang^1^, Chung-Ping Chang^1^and Shyi-Dong Yeh^1,2,3^*

^1^Department of Plant Pathology, National Chung Hsing University, Taichung, Taiwan, R.O.C.

^2^NCHU-UCD Plant and Food Biotechnology Center, National Chung Hsing University, Taiwan, R.O.C.

^3^Department of Chinese Pharmaceutical Sciences and Chinese Medicine Resources, China Medical University, Taichung, Taiwan, R.O.C.

^4^Agricultural Biotechnology Center, National Chung Hsing University, Taichung, Taiwan, R.O.C.

***Corresponding author: Shyi-Dong Yeh**

**Phone: 886-4-22877021**

**Fax: 886-4-22852501**

**E-mail:** [**sdyeh@nchu.edu.tw**](mailto:sdyeh@nchu.edu.tw)

File includes: Figures: S1-S2

Tables: S1-S4

**SUPPLEMENTARY DATA**

**Table S1.** Nucleotide (in upper corner) and amino acid (in lower corner) sequences of the HC-Pro coding region of the super strain 5-19 of *Papaya ringspot virus* (PRSV) were compared with those of other geographic strains from Taiwan (YK), Thailand (TH), Hawaii (HA), and Mexico (MX).

| Virus strain^a^ | 5-19 | YK | TH | HA | MX |
| --- | --- | --- | --- | --- | --- |
| 5-19 | … | 96.8^b^ | 91.1 | 86.8 | 86.1 |
| YK | 98.7^c^ | … | 91.0 | 86.5 | 85.8 |
| TH | 96.5 | 96.1 | … | 86.1 | 91.8 |
| HA | 95.8 | 95.6 | 95.2 | … | 84.7 |
| MX | 95.0 | 95.0 | 96.1 | 94.3 | … |

^a^Sequences of HC-Pro from YK^40^, and HA^43^ and 5-19, MX and TH (this study) were used for analysis. Sequence comparison was conducted with PC/GENE software (version 6.85, IntelliGenetics, Inc., Mountain View, CA). ^b^Nucleotide sequence identity. ^c^Amino acid sequence identity.

**Table S2.** Resistance evaluation of transgenic papaya lines carrying individual untranslatable HC-Pro constructs of *Papaya ringspot virus* (PRSV) 5-19 by mechanical inoculation with CP-transgenic-resistance-breaking strain 5-19 or non-breaking strain YK

| Construct | No. of assayed lines | |  | Response after challenge with ^a^ | | | | | | | | | | | | | |
| --- | --- | --- | --- | --- | --- | --- | --- | --- | --- | --- | --- | --- | --- | --- | --- | --- | --- |
|  |  |  |  | PRSV 5-19 | | | | | | |  | PRSV YK | | | | | |
|  |  |  |  | Highly resistant^b^  (HR％) | | | Weakly resistant | | Susceptible | |  | Highly resistant  (HR％) | | Weakly resistant | | Susceptible | |
| pBI-519HCF | 15 |  |  | | 7^c^ | (46.6) | 4 |  | 4 |  |  | 8 | (53.3) | 3 |  | 4 |  |
| pBI-519HCN | 31 |  |  | | 9 | (29.0) | 2 |  | 20 |  |  | 11 | (35.5) | 1 |  | 19 |  |
| pBI-519HCC | 14 |  |  | | 3 | (21.4) | 4 |  | 7 |  |  | 4 | (28.6) | 3 |  | 7 |  |
| **Total** | **60** |  |  | | **19** | **(31.6)** | **10** |  | **31** |  |  | **23** | **(36.7)** | **7** |  | **30** |  |

^a^Five plants of each line or control were mechanically inoculated with PRSV 5-19 or PRSV YK. Results were recorded 28 days post inoculation (dpi).

^b^Susceptible lines showed mosaic symptoms on leaves after inoculation with PRSV 5-19 or YK at14 dpi. Weakly resistant lines showed a 1-2 week delay in symptom development compared with controls. Highly resistant lines showed no symptoms and were ELISA negative at 28 dpi using PRSV antiserum. PRSV YK CP-3'UTR transgenic papaya lines 18-2-4 highly resistant to PRSV YK^21^, but susceptible to PRSV 5-19^24^, and non-transgenic papaya (NT) were used as controls. All ten NT plants were susceptible to YK and 5-19; all ten 18-2-4 plants were resistant to YK but susceptible to 5-19 in this experiment.

^c^Numbers of lines (％ of the total tested lines). All PRSV 5-19 resistant or highly resistant lines were also highly resistant to PRSV YK.

**Table S3.** Responses of R_0_ plants of selected papaya transgenic lines carrying untranslatable a full-length (F lines) or partial (N lines) sequence of the silencing suppressor HC-Pro from the super *Papaya ringspot* *virus* (PRSV) strain 5-19, all with only a single transgene insert, after mechanical inoculation with five PRSV strains originating from different geographic regions

| Line | Dpi | Number^a^ of plants with symptoms after inoculation | | | | |
| --- | --- | --- | --- | --- | --- | --- |
|  |  | 5-19 | YK | MX | TH | HA |
| NT |  |  |  |  |  |  |
|  | 14 | 5 | 5 | 5 | 5 | 5 |
| 18-2-4 |  |  |  |  |  |  |
|  | 14 | 5 | 0 | 0 | 0 | 0 |
|  | 28 | 5 | 0 | 3 | 2 | 3 |
|  | 42 | 5 | 0 | 5 | 3 | 4 |
| F2-1-4 |  |  |  |  |  |  |
|  | 14 | 0 | 0 | 4 | 0 | 0 |
|  | 28 | 0 | 0 | 5 | 1 | 1 |
|  | 42 | 0 | 0 | 5 | 4 | 4 |
| F2-7-1 |  |  |  |  |  |  |
|  | 14 | 0 | 0 | 5 | 5 | 2 |
|  | 28 | 0 | 0 | 5 | 5 | 3 |
|  | 42 | 0 | 0 | 5 | 5 | 4 |
| N11-1 |  |  |  |  |  |  |
|  | 14 | 0 | 0 | 5 | 0 | 0 |
|  | 28 | 0 | 0 | 5 | 2 | 3 |
|  | 42 | 0 | 0 | 5 | 5 | 5 |
| F3-2-2 |  |  |  |  |  |  |
|  | 14 | 0 | 0 | 0 | 0 | 0 |
|  | 28 | 0 | 0 | 0 | 0 | 0 |
|  | 42 | 0 | 0 | 0 | 0 | 0 |

^a^Numbers of plants showing symptoms from the five plants tested for each line. NT: non-transgenic control. Strains from Taiwan (YK), Hawaii (HA), Thailand ( TH), and Mexico (MX) were used for mechanical inoculation. Responses of the plants were recorded at 14, 28, and 42 days after inoculation.

**Table S4.**  Segregation ratios of R_1_ progenies from the self-pollinated HC-Pro transgenic papaya R_0_ lines F2-1-4 and F3-2-2, as determined by kanamycin resistance and bioassay

| R_1_ progeny of  Transgenic line | Insert number^a^ | Number of premature seeds | | | | Segregation ratio | Predicted no. of *npt*II loci | *χ*^2^ | *P*^b^ |
| --- | --- | --- | --- | --- | --- | --- | --- | --- | --- |
|  |  | Resistant^c^ | | Sensitive | |  |  |  |  |
| F2-1-4 | 1 | 88 |  | 29 |  | 3:1 | 1 | 0.003 | 0.957 |
| F3-2-2 | 1 | 77 |  | 25 |  | 3:1 | 1 | 0.013 | 0.909 |
| Tainung No. 2 | － | 0 |  | 50 |  | － | － | － | － |

^a^The insert numbers of the transgene in the two lines are shown in Fig. S2.

^b^Probability of goodness of fit was set at a significance level of 0.05.

^c^All kanamycin-resistant plants were resistant to PRSV 5-19 in bioassays.


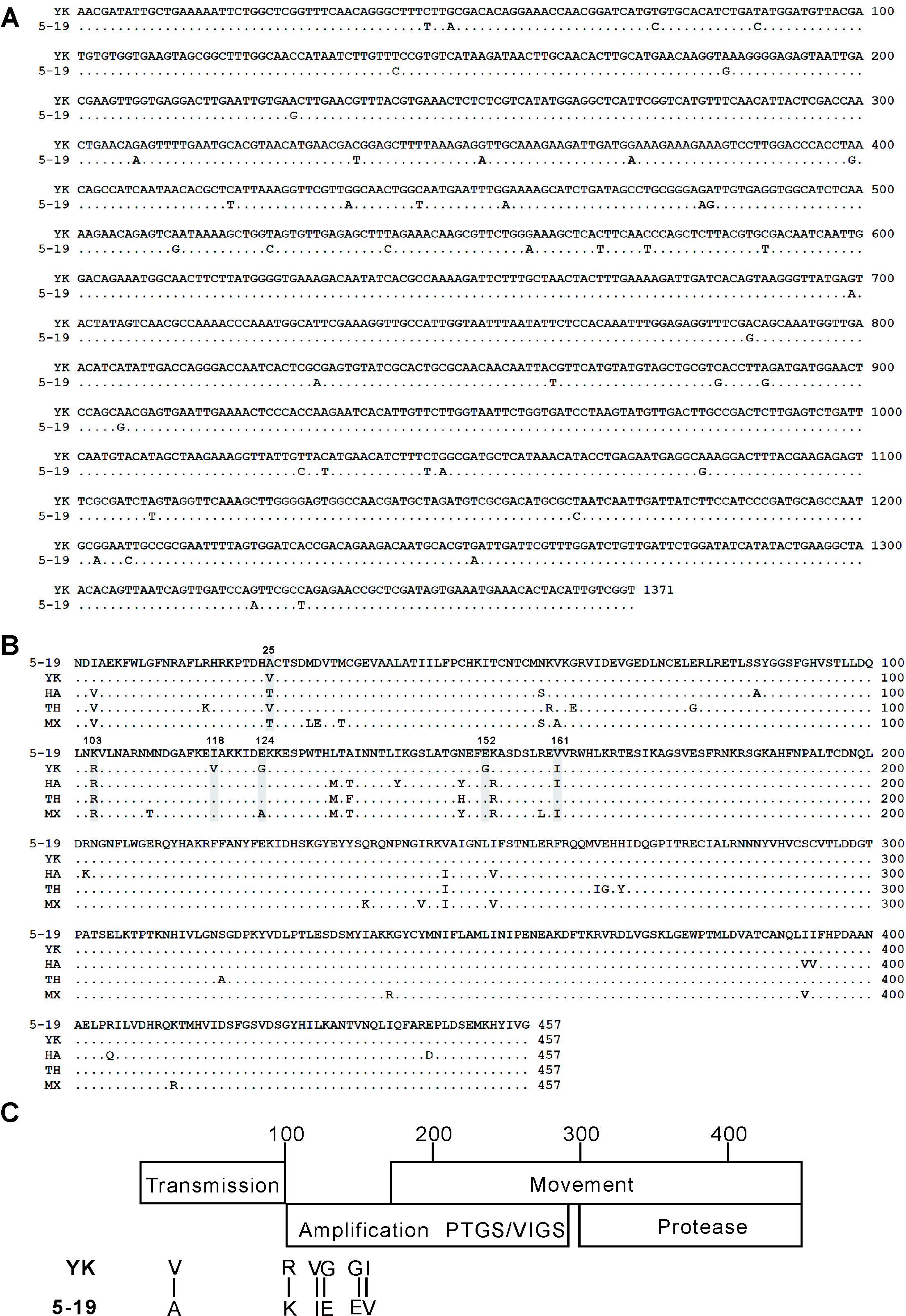


**Figure S1** Comparison of the coding sequence of the helper component protease (HC-Pro) of *Papaya ringspot virus* (PRSV) 5-19 with those of other PRSV strains. **(A)** Comparison of nucleotide sequences of the HC-Pro coding sequence of PRSV 5-19 with that of PRSV YK. Dots (“.”) indicate aa identities in the aligned sequences. **(B)** Alignment of the amino acid sequence of the HC-Pro of PRSV 5-19 with those of four other PRSV strains of different geographic origins; dots (“.”) indicate aa identities in the aligned sequences, whereas single letter codes indicate aa variations. The aa differences between 5-19 and YK are shown in grey boxes. The sources of these PRSV strains were described in “Materials and Methods”. Sequence alignments generated using the PILEUP program of the GCG package (version 9.0, Genetics Computer Group, Madison, WI). **(C)** Biological functions of different HC-Pro regions^38^ and the positions of amino acid differences between YK and 5-19.


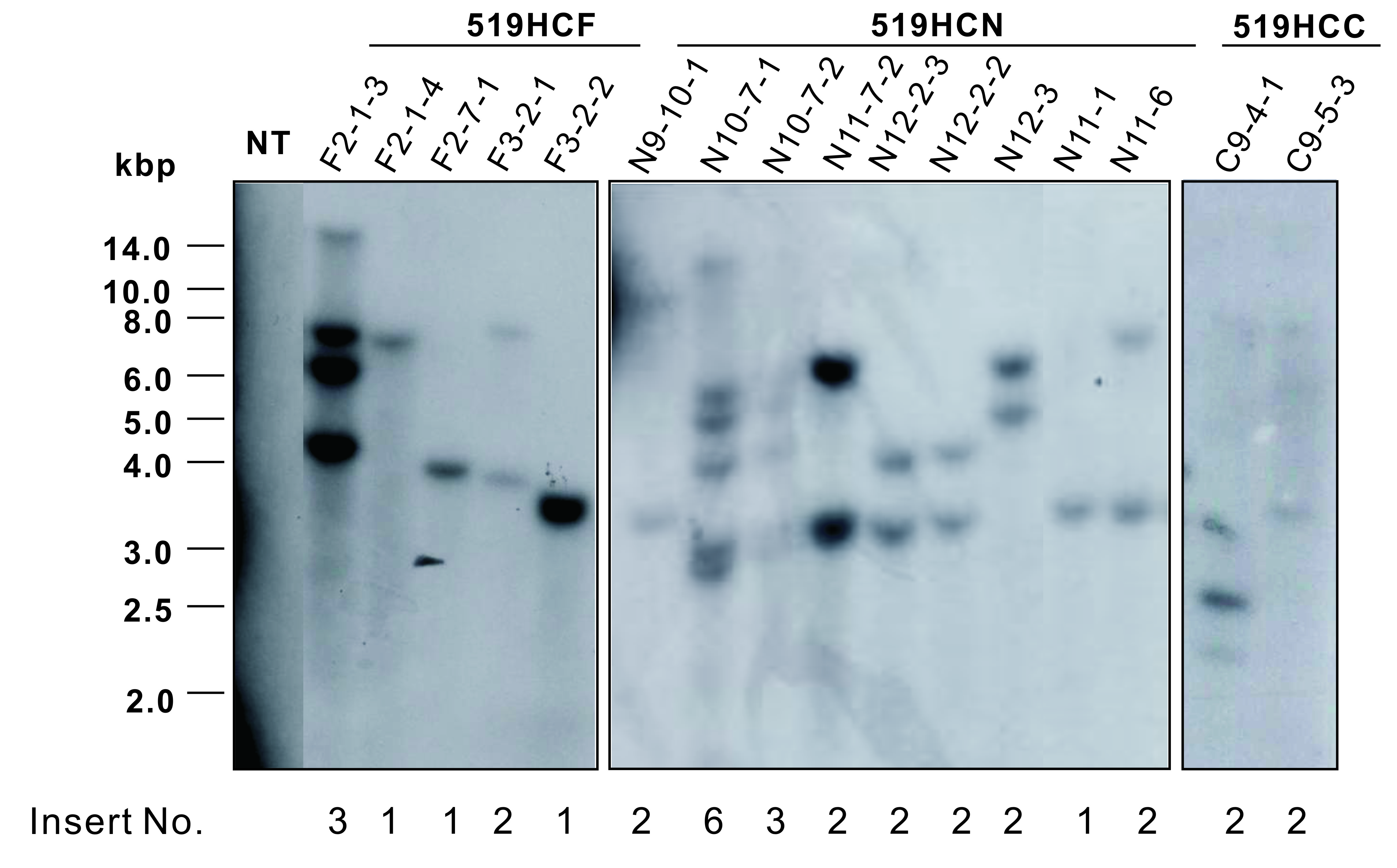


**Figure S2** Transgene insertion of R_0_ transgenic papaya lines carrying untranslatable full-length (519HCF), N-terminal (519HCN), or C-terminal (519HCC) HC-Pro were analysed by Southern hybridisation. The insert numbers (Insert No.) are indicated at the bottom of each lane. Non-transgenic papaya (NT) was used as a negative control. For hybridisation, electrophoresis and blotting of all the DNA samples were conducted under uniform conditions and blots were hybridised in a single reaction.
